# Evaluation of Altered Glutamatergic Activity in a Piglet Model of Hypoxic-Ischemic Brain Damage Using ^1^H-MRS

**DOI:** 10.1155/2020/8850816

**Published:** 2020-09-23

**Authors:** Yuxue Dang, Xiaoming Wang

**Affiliations:** Department of Radiology, Shengjing Hospital of China Medical University, Shenyang 110004, China

## Abstract

**Methods:**

Twenty-five newborn piglets were selected and then randomly assigned to the control group (*n* = 5) and the model group (*n* = 20) subjected to HI. HI was induced by blocking bilateral carotid blood flow under simultaneous inhalation of a 6% oxygen mixture. ^1^H-MRS data were acquired from the basal ganglia at the following time points after HI: 6, 12, 24, and 72 h. Changes in protein levels of EAAT2 and GluR2 were determined by immunohistochemical analysis. Correlations among metabolite concentrations, metabolite ratios, and the protein levels of EAAT2 and GluR2 were investigated.

**Results:**

The Glu level sharply increased after HI, reached a transient low level of depletion that approached the normal level in the control group, and subsequently increased again. Negative correlations were found between concentrations of Glu and EAAT2 protein levels (*R*_s_ = −0.662, *P* < 0.001) and between the Glu/creatine (Cr) ratio and EAAT2 protein level (*R*_s_ = −0.664, *P* < 0.001). Moreover, changes in GluR2 protein level were significantly and negatively correlated with those in Glu level (the absolute Glu concentration, *R*_s_ = −0.797, *P* < 0.001; Glu/Cr, *R*_s_ = −0.567, *P* = 0.003).

**Conclusions:**

Changes in Glu level measured by ^1^H-MRS were inversely correlated with those in EAAT2 and GluR2 protein levels following HI, and the results demonstrated that ^1^H-MRS can reflect the early changes of glutamatergic activity in vivo.

## 1. Introduction

The excitatory amino acid glutamate (Glu) is a major excitatory neurotransmitter in the central nervous system of mammals and has a crucial role in maintaining normal brain function. Under normal physiological conditions, the Glu level in extracellular fluid is only 0.5-5 *μ*M [1]. Astrocytes maintain a low Glu level in extracellular fluid and prevent Glu excitotoxicity and abnormal synaptic transmission. Glial fibrillary acidic protein (GFAP) is widely recognized as a specific molecular marker of astrocytes, as it is the protein mostly related to astrocytic functions [2, 3]. The excitatory amino acid transporter 2 (EAAT2, also called glutamate transporter 1 (GLT-1) in rodents) on the astrocyte cell membrane is responsible for the majority of Glu transport in the body [4, 5]. Glu released by presynaptic neurons enters into astrocytes by reuptake and is transformed into the nonexcitatory amino acid glutamine (Gln) by glutamine synthetase, which subsequently undergoes uptake by presynaptic neurons to complete the Glu-Gln cycle [6].

Exposure to hypoxia-ischemia (HI) injury induces substantial Glu release from presynaptic neurons [7] and impairs the activity of Glu reuptake systems. Consequently, excessive Glu accumulate in the synaptic spaces and bind with glutamate receptors located on the postsynaptic neural membranes, which results in excitotoxicity. The main excitatory ionotropic glutamate receptors are the *N*-methyl-D-aspartate acid receptor (NMDAR) [8] and the *α*-amino-3-hydroxy-5-methyl-4-isoxazole-proprionic acid receptor (AMPAR) [9]. The NMDAR and AMPAR can be activated by excess Glu, and they have crucial roles in mediating Glu excitotoxicity [8–10]. Most studies have focused on determining the mechanism of NMDAR mediation of Glu excitotoxicity in hypoxic-ischemic brain damage (HIBD). Subsequent research proposed the “GluR2 hypothesis” [9], which considers that HI-induced structural changes in AMPAR mediate neuronal injury. The GluR2 subunit is an important functional moiety of the AMPAR; GluR2 determines the Ca^2+^ permeability of AMPAR [11–13] and is involved in mediating Glu excitotoxicity.

The occurrence of HIBD in perinatal newborns generally injures specific brain regions. The deep gray matter nuclei are very easily injured by HI [14], and the basal ganglia are highly susceptible to Glu excitotoxicity [15]. The immature brain of infants is more susceptible to Glu excitotoxicity than the mature adult brain [14–16]. HIBD is an important cause of permanent dysfunction and death of perinatal newborns, occurring approximately 2-3 per 1000 term births [17], which affects both the families and society. No therapeutic strategies have been developed to effectively improve the quality of life and the survival of these patients.

The present study was aimed at investigating the glutamate metabolism alterations in the basal ganglia using ^1^H-MRS following HI insult in a piglet model and preliminarily exploring the possible mechanisms of Glu excitotoxicity.

## 2. Materials and Methods

### 2.1. Experimental Animals

All animal experiments were performed in accordance with the Regulations for the Administration of Affairs Concerning Experimental Animals (http://www.asianlii.org/cn/legis/cen/laws/rftaoacea704/). The protocol was approved by the Animal Ethics Committee of Shengjing Hospital of China Medical University, Shenyang, China. Twenty-five newborn male Yorkshire piglets (P3-5, body weight: 1.5-2.0 kg) were selected from the Laboratory Animal Center and then randomly assigned to the control group (sham-operation group, *n* = 5) and the HI model group (*n* = 20). The HI model group was allocated into four subgroups with differing assessment times after HI-induced brain injury: 6, 12, 24, and 72 h (*n* = 5 piglets per group). The experimental animals were maintained with unlimited food and water in a quiet and warm environment.

### 2.2. Preparation of Animal Models

The newborn piglets in the model group were initially anesthetized with intramuscular injection of 0.6 mL/kg xylazine hydrochloride. After anesthesia, the animals were fixed on the operation bench in a supine position. Heating pads were employed during surgery to maintain body temperature at 37 ± 0.5°C. Tracheal intubation (*φ* 2.5 mm) was performed, and then, each piglet was connected to a TKR-200C small animal ventilator for mechanical ventilation with 100% oxygen and the following ventilator parameters: inspiration/expiration (*I*/*E*) = 1 : 1.5 (respiration ratio) and respiration rate = 30/min. The heart rate and oxygen saturation of blood were monitored continuously using a TuffSat handheld pulse oximeter (GE Healthcare, Milwaukee, Wisconsin, USA). The incision site and adjacent skin were disinfected, and the piglets were subjected to a middle anterior neck incision. Bilateral common carotid arteries were isolated from adjacent internal jugular veins and vagus nerves. After the condition of the animal was stable for 40 min, the bilateral common carotid arteries were occluded using small arterial clamps. Then, a gas mixture containing 6% oxygen and 94% nitrogen was inhaled mechanically for 40 min. After 40 min, the small artery clamps on the bilateral common carotid arteries were removed, and blood flow was recovered. Simultaneously, oxygen (100%) was mechanically inhaled again, and the incision was stitched. After the operation, each piglet was transferred to an incubator (37°C) to maintain normal body temperature during postsurgical recovery. Piglets in the control group (pseudooperation group) underwent the same presurgical preparation as those in the model group but were not subjected to the HI induction procedures.

All operations were conducted under effective analgesia and anesthesia to reduce animal suffering.

### 2.3. Magnetic Resonance Imaging

The MRI examination was conducted for all animals in both the sham-operation and model groups. The piglets were anesthetized with xylazine hydrochloride. Then, the animals were placed in a supine position with a special foam pad around their heads to keep the head centered. Then, the following scans were performed: conventional fast-field echo (FFE) T1-weighted imaging (T1WI) (repetition time (TR)/echo time (TE), 200/2.3 ms; matrix, 224 × 162; and slice thickness, 5 mm) and turbo spin-echo (TSE) T2-weighted imaging (T2WI) (TR/TE, 5000/80 ms; matrix, 224 × 162; and slice thickness, 5 mm). MRI scans were performed using the Philips Achieva 3.0T MRI system (Best, Netherlands) with 8-channel phase array head coils. The newborn piglets were also carefully wrapped in thick quilts to maintain temperature.

^1^H-MRS scans were performed using a point-resolved spectroscopy (PRESS) sequence for single-voxel acquisition (TR, 2000 ms; TE, 37 ms; samples, 1024; bandwidth, 2000 Hz; and NSA, 64). A short TE sequence (TE = 37 ms) was utilized for better demonstration of the Glu peak, which could reduce the impact of relaxation effect, obtaining a better spectrum. Automatic shimming was completed before scanning. The location was determined at the level of the basal ganglia by axial T2WI, and the volume of interest (VOI) of 10 × 10 × 10 mm was placed in the left basal ganglia. Care was taken to avoid noise caused by the surrounding areas, such as cerebrospinal fluid, blood vessels, fat, and air. The saturation band was placed at an area outside the VOI, and field shimming and water-suppressing operations were performed within the VOI, achieving full width at half maximum (FWHM) ≤ 10 Hz and water suppression > 98% and allowing subsequent collection of spectral data. The basal ganglia were selected as the region of interest (ROI) because they are one of the most susceptible regions to HIBD in newborns.

### 2.4. ^1^H-MRS Postprocessing and Data Analysis

Spectral raw data obtained by ^1^H-MRS scanning were quantitatively analyzed using linear combination model software (LCModel, version 6.3-1B, S.W. Provencher) [18]. This popular software for quantitative analysis of spectral data employs a black box operation that allows automatic averaging of the original spectral images, baseline correction and smoothing, phase correction, metabolite identification, and finally acquisition of data for different metabolites. The absolute quantities of metabolites were obtained, and the Cramér-Rao lower bounds (CRLBs) were calculated; these were used as an index of metabolite quantification to evaluate the reliability of the fitted results. Spectra were fitted with a chemical shift at approximately 0.2-4.0 ppm (ppm = 10^−6^) using the LCModel software. The final simulated spectra included the following 17 metabolites: alanine (Ala), aspartate (Asp), creatine (Cr), phosphocreatine (PCr), *γ*-aminobutyric acid (GABA), glucose (Glc), Glu, Gln, glycerophosphorylcholine (GPC), phosphorylcholine (PCho), glutathione (GSH), inositol (Ins), lactic acid (Lac), *N*-acetylaspartate (NAA), *N*-acetylaspartylglutamate (NAAG), scyllitol (Src), and taurine (Tau). The baseline setting of the basic set also included macromolecules and lipids. Only the spectrum data with CRLBs < 50% and generally <25% and signal‐to‐noise ratio (SNR) ≥ 5 were included in the statistical analysis.

We analyzed the levels of Glu, Gln, Glx (Glu+Gln complex), NAA (NAA+NAAG), choline-containing compounds (Cho) (GPC+PCho), and Cr (Cr+PCr), and the total amounts were used for NAA, Cho, and Cr to guarantee the reliability of data. Glu generates complex signals at approximately 2.04-2.35 ppm and 3.75 ppm with a prominent peak at 2.35 ppm. Neurotoxicity occurs when the Glu content exceeds the physiological demand of Glu for neurotransmission. Gln, which is an intermediate metabolite supporting multiple pathways of energy metabolism and neurological transmission, forms resonance peaks at 2.45, 3.78, and 2.15 ppm. Although the J-coupling effect between Glu and Gln causes their peaks to overlap with each other, we found that the software could correctly separate them to a certain extent. Therefore, qualitative analysis was carried out for Glu and Gln individually. The main NAA peak is at 2.02 ppm, which reflects the mitochondrial functions of neurons [19]. Cho is an important cell membrane phospholipid, and its main peak is at 3.20 ppm. The predominant Cr peaks are at 3.03 and 3.94 ppm; Cr is important for energy metabolism in neurons and the astrocyte cytoplasm.

In this study, the absolute concentrations of Glu, Gln, Glx, NAA, Cho, and Cr in the basal ganglia were analyzed. Additionally, we measured the Glu/Cr, Gln/Cr, Glx/Cr, NAA/Cr, and Cho/Cr concentration ratios (namely, the relative concentration) which were also provided by the LCModel software.

### 2.5. Histological Examination

After the MRI examination was completed at the specified time points, the newborn piglets were immediately sacrificed and their brains were rapidly collected for pathological examination. The brains were fixed in 10% formaldehyde solution for 48 h and then sectioned at a coronal plane. Then, sections containing the basal ganglia were embedded in paraffin and thin-sectioned with 4 *μ*m thickness for conventional hematoxylin-eosin (HE) and immunohistochemical (IHC) staining.

The HE-stained brain sections were evaluated under a light microscope for pathological changes in the basal ganglia. The brain changes were assessed and scored with reference to the brain pathological evaluation standards of Li et al. [20]: a score of 0-6 for nervous pathological injury, with 0-3 for cerebral edema (0, none; 1, mild; 2, moderate; and 3, severe) and 0-3 for nerve cell injury and necrosis (0, none; 1, mild; 2, moderate; and 3, severe). The total score was the sum of individual scores, and a higher total score indicated more severe injury. Brain edema includes cytotoxic edema and vasogenic edema. Cell necrosis includes the death of individual cells, groups of cells, and all cells in a certain region. The evaluation was conducted by an experienced professional physician who was blinded to the experimental grouping, and it was based on the observation of cell morphological changes under the light microscope (400x magnification).

IHC staining procedures were as follows. The paraffin sections were incubated with 3% H_2_O_2_ at room temperature for 15 min to block endogenous peroxidase activity and then blocked with 5% normal goat serum at room temperature for 30 min. Thereafter, these sections were incubated overnight at 4°C with the following primary antibodies: rabbit anti-GFAP (1: 1000, Abcam), rabbit anti-EAAT2 (1 : 200, Abcam), and mouse anti-GluR2 (1 : 100, Abcam). Then, the sections were washed, incubated with biotinylated anti-rabbit/mouse immunoglobulin G at 37°C for 1 h, developed with DAB, counterstained with hematoxylin, and mounted with neutral balsam. The prepared sections were observed under light microscopy for staining of the basal ganglia. Phosphate-buffered saline was used instead of primary antibodies for negative controls, and other procedures were the same. After the addition of the secondary antibodies, all procedures were performed while protecting the sections from light. All images were analyzed with the image analysis system. Five fields (400x magnification) were randomly selected to measure the optical density of antibody binding, and the mean optical density (OD) was used as the measured value (arbitrary units) of GFAP, EAAT2, and GluR2 expression.

### 2.6. Statistical Analysis

The homogeneity of data variance was analyzed by the Levene test. The homogeneity of variance determined via multigroup comparison was analyzed by one-way ANOVA. The heterogeneity of variance was analyzed by Welch's *t*-test. The categorical data was analyzed by the Kruskal-Wallis test. Correlations between spectral data and pathological results were analyzed using the Spearman correlation analysis with *R*_s_ as the correlation coefficient. SPSS v. 20.0 statistical software (IBM, NY, USA) was used for all analyses. All statistical tests were two-tailed, with *P* < 0.05 considered statistically significant.

## 3. Results

### 3.1. HI-Induced Changes in Neuron and Astrocyte Morphologies in the Basal Ganglia

**Figure 1:**
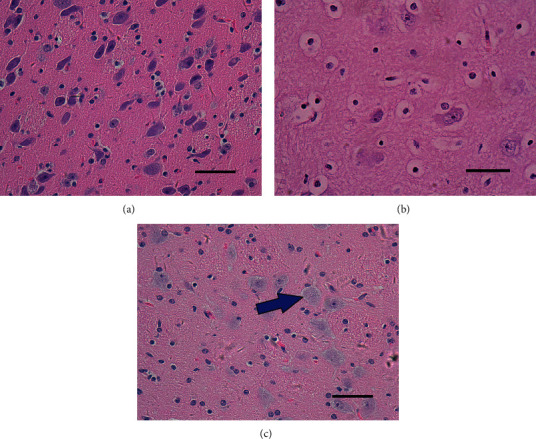
Typical images of hematoxylin and eosin staining of the piglet basal ganglia (400x magnification). Scale bar = 50 *μ*m. (a) In the control group, piglet nerve cells were regularly arranged with normal morphology. (b, c) At the later stage following HI, piglet nerve cells were significantly swollen, the cells were slightly stained, the intracellular space was widened, and karyolysis (arrow) was observed.

**Figure 2:**
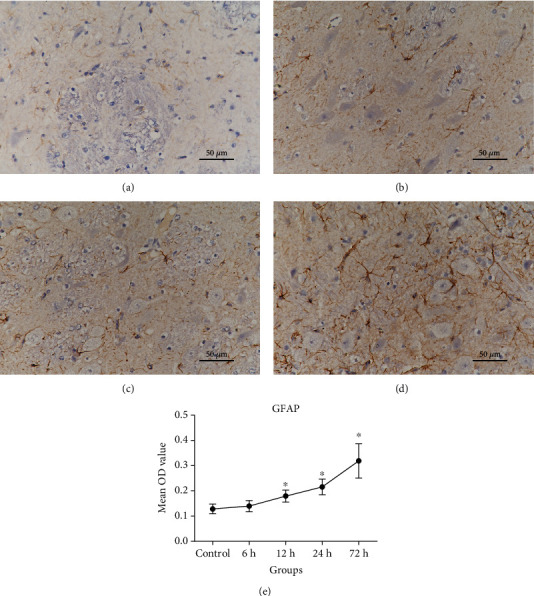
Changes in GFAP expression in the basal ganglia of piglets (400x magnification). Scale bar = 50 *μ*m. (a–d) Representative figures of GFAP IHC staining in the control group and HI insult 12 h, 24 h, and 72 h subgroups. (e) Changes in the average OD of GFAP protein level. Compared with the control group, the expression levels of the GFAP were significantly increased in the HI group. GFAP: glial fibrillary acidic protein; IHC: immunohistochemical; OD: optical density. Error bars represent the standard deviation values (*n* = 5/group). ^∗^*P* < 0.05 compared with the control group.

In the control group, neurons were regularly arranged, with normal cell morphology, rich cytoplasm, and clear nuclei. Astrocytes have low GFAP-positive response, light staining, small cell volume, slender and short protrusions, and sparse distribution. However, in the HI model group, the number of GFAP-positive cells increased, the staining was deep, the cell body was large, and the protrusions grew thick. The HI-treated neurons and astrocytes displayed the following changes at specific time points: at 6 h after HI, neurons did not display any significant morphological changes; at 12 h, many astrocytes were swollen and displayed a lightly stained cytoplasm and vacuoles; at 24 h, many neurons and astrocytes were swollen; and at 72 h, astrocytes were clearly swollen and degenerated, the neuronal cell membrane was damaged, and nuclei were swollen and lightly stained (Figures 1 and 2). The pathological damage of brain tissues became more severe over time. The pathological scores at various time points are presented in Table 1.

### 3.2. HI-Induced Changes in GFAP, EAAT2, and GluR2 Expression in the Basal Ganglia

GFAP, as a biomarker protein of astrocytes, changed significantly after HI. The results showed an increase in expression levels of GFAP immunostaining observed after HI in comparison to the control group. And there were statistically significant differences in HI insult 12 h, 24 h, and 72 h subgroups with respect to the control group (both *P* < 0.05) (Figure 2).

**Figure 3:**
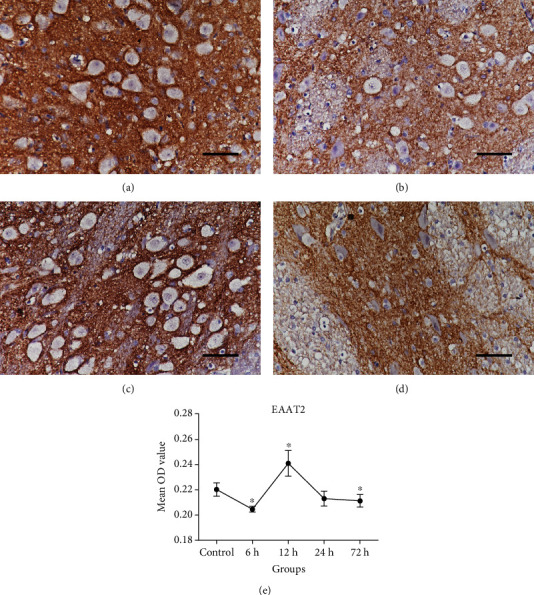
EAAT2 protein levels in the basal ganglia before (control) and after HI injury (400x magnification). Scale bar = 50 *μ*m. (a–d) Representative figures of EAAT2 IHC staining in the control group and in 6 h, 12 h, and 72 h HI subgroups. (e) Changes in the average OD of EAAT2 protein. During early HI, EAAT2 protein level slightly decreased compared with the control; subsequently, it tended to increase and then decrease over time after HI injury. EAAT2: excitatory amino acid transporter 2; IHC: immunohistochemical; OD: optical density. Error bars represent the standard deviation values (*n* = 5/group). ^∗^*P* < 0.05 compared with the control group.

In the normal control group, EAAT2 was mainly expressed in the plasma membranes of cells. HI caused significant changes in the expression of EAAT2. The EAAT2 expression level in the basal ganglia was significantly lower in the HI insult 6 h subgroup than in the control group (*P* = 0.001). Then, EAAT2 expression tended to markedly increase in the 12 h subgroup and thereafter decreased again (Figure 3). There was a statistically significant difference between EAAT2 expression in the 12 h subgroup, the 72 h subgroup, and the control group (*P* < 0.001 or *P* = 0.033).

**Figure 4:**
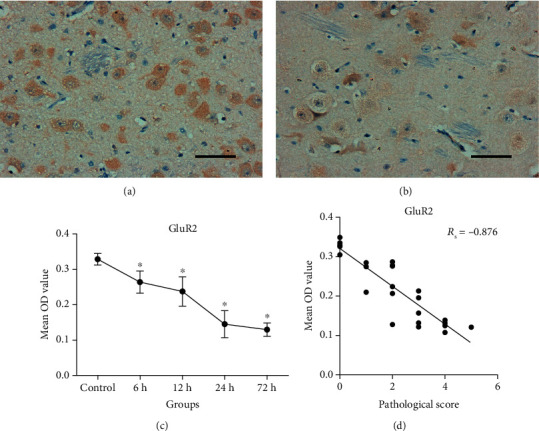
AMPAR subunit GluR2 protein levels in the basal ganglia before (control) and after HI injury (400x magnification). Scale bar = 50 *μ*m. (a) High GluR2 protein levels in the basal ganglia in the control group. (b) At 72 h after HI injury, the GluR2 protein level was markedly reduced. (c) Changes in the average OD of GluR2 protein level visualized with IHC staining. GluR2 protein level declined in piglets subjected to HI injury compared with the control. (d) GluR2 protein level was negatively correlated with the severity of pathological lesions; i.e., the more severe the brain injury, the more obvious the downregulation of GluR2 protein level. AMPAR: *α*-amino-3-hydroxy-5-methyl-4-isoxazole-proprionic acid receptor; IHC: immunohistochemical; OD: optical density. Error bars represent the standard deviation values (*n* = 5/group). ^∗^*P* < 0.05 compared with the control group. The Spearman rank correlation coefficient was presented as *R*_s_.

The GluR2 protein level in the basal ganglia decreased significantly over time after HI compared with that in the control, and the differences were statistically significant (both *P* < 0.05) (Figure 4). The results also indicated that the GluR2 protein level was negatively correlated with the severity of pathological lesions (*R*_s_ = −0.876, *P* < 0.001) and the GluR2 expression was lower when HI-induced brain damage was more severe.

### 3.3. ^1^H-MRS Results

**Figure 5:**
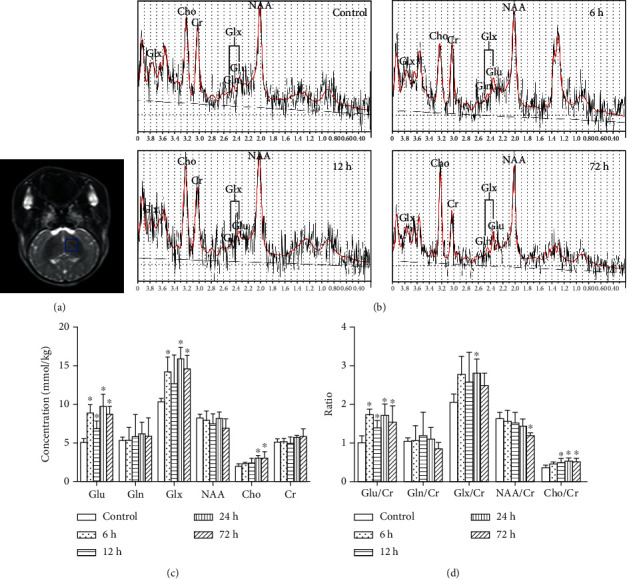
Representative ^1^H-MRS images of the basal ganglia of piglets at different time points after HI and changes in metabolite absolute concentrations and ratios. (a) Axial T2-weighted MR image obtained from a control piglet; the blue box indicates the voxel location. (b) The following metabolite peaks were observed: Glu, Gln, Glx, NAA, Cho, and Cr. (c, d) Changes in metabolite absolute concentrations and metabolite ratios in the control and HI-induced groups at different time points are shown. Glu: glutamate; Gln: glutamine; Glx: glutamate/glutamine complex; NAA: *N*-acetylaspartate; Cho: choline; Cr: creatine. Error bars represent the standard deviation values (*n* = 5/group). ^∗^*P* < 0.05 compared with the control group.

The results in Figure 5 showed the changes of metabolites by ^1^H-MRS. Compared with the control group, the absolute Glu concentrations markedly changed over time after HI insult (Figure 5(c)). The Glu levels showed a biphasic change. The Glu concentrations clearly increased at 6 h after HI treatment and then reached a transient minimum at 12 h that was approaching the level in the control group and then increased again at 24 h. There were statistically significant differences in Glu concentrations between the various groups (*F* = 14.781, *P* < 0.001). The intergroup analysis indicated that there were significant differences between the control and HI groups at 6, 12, 24, and 72 h after HI injury (*P* < 0.001, *P* = 0.017, *P* < 0.001, and *P* < 0.001, respectively) and between HI groups at 12 h versus 6, 24, and 72 h (*P* = 0.008, *P* = 0.001, and *P* = 0.013), but there were no significant differences between other subgroups. The trend of the absolute concentration of Glx was similar to that of Glu, which was significantly elevated at 6 h, 24 h, and 72 h after HI when compared with the control group (both *P* < 0.05). The Cho concentrations appeared to increase over time after the HI insult. There were significant differences between the 24 h and 72 h HI subgroups and the control group (*P* = 0.006 and *P* = 0.006, respectively). Moreover, significant correlations were found between Cho concentrations and the pathological scores (*R*_s_ = 0.703, *P* < 0.001). While there were no differences in the absolute concentrations of Gln, NAA or Cr was observed between the different groups (*F* = 0.360, *P* > 0.05; *F* = 1.382, *P* > 0.05; and *F* = 1.965, *P* > 0.05).

Changes in Glu/Cr and Glx/Cr ratios were similar to the changes in the Glu or Glx concentrations (Figure 5(d)). This study also analyzed the changes in NAA/Cr and Cho/Cr ratios. NAA/Cr gradually declined over time after HI insult, with statistically significant differences observed between the 72 h HI subgroup and the control group (*P* = 0.003). The results also indicated that changes in NAA/Cr were negatively correlated with the severity of pathological lesions in the basal ganglia (*R*_s_ = −0.456, *P* = 0.022). There was a similar change in Cho/Cr concentration ratios with the absolute Cho concentrations. The increase in Cho/Cr ratios in the 12 h, 24 h, and 72 h HI subgroups was considered to have statistically significant difference as compared with the control group (*P* = 0.020, *P* = 0.004, and *P* = 0.010, respectively). And the Cho/Cr ratio showed a significant correlation with the pathological scores (*R*_s_ = 0.638, *P* = 0.001).

### 3.4. Correlations among HI-Induced Changes in GluR2, EAAT2, and Glu Levels in the Basal Ganglia

**Figure 6:**
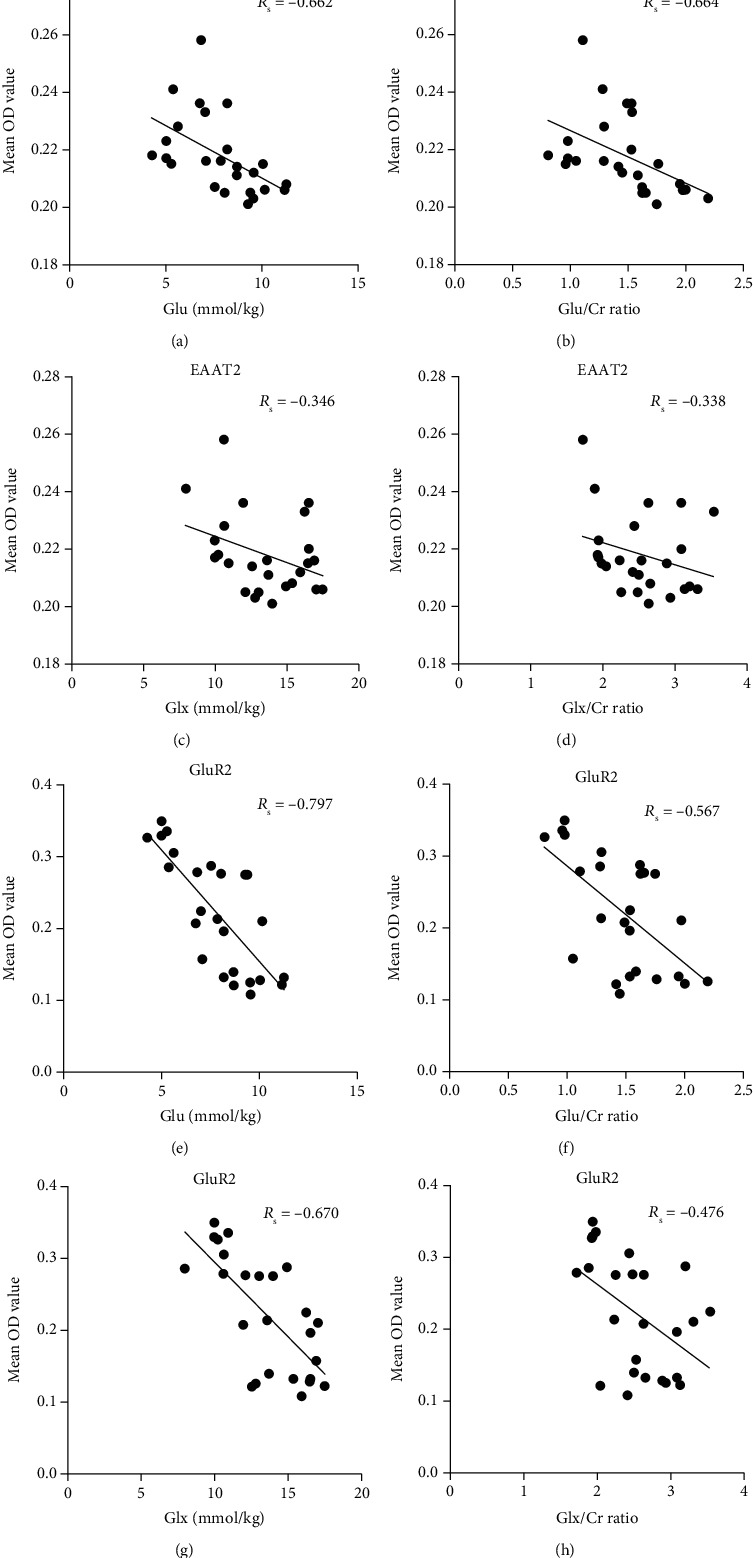
Scatter plot of correlations between the expression levels of EAAT2 or AMPAR subunit GluR2 protein levels and the Glu or Glx metabolite levels in the basal ganglia of piglets. EAAT2: excitatory amino acid transporter 2; AMPAR: *α*-amino-3-hydroxy-5-methyl-4-isoxazole-proprionic acid receptor; OD: optical density; Glu: glutamate; Glx: glutamate/glutamine complex. The Spearman rank correlation coefficient was presented as *R*_s_.

After HI insult, the changes in Glu concentrations and the dynamic changes in EAAT2 expression were significantly negatively correlated in the basal ganglia (*R*_s_ = −0.662, *P* < 0.001), and changes in the Glu/Cr ratios were significantly negatively correlated with EAAT2 expression (*R*_s_ = −0.664, *P* < 0.001) (Figures 6(a) and 6(b)). However, there was no significant correlation between the absolute concentration of Glx, the Glx/Cr ratio, and the expression of EAAT2 (*R*_s_ = −0.346, *P* > 0.05; *R*_s_ = −0.338, *P* > 0.05) (Figures 6(c) and 6(d)).

Moreover, the absolute Glu concentrations and GluR2 protein expression level in the basal ganglia were significantly correlated (*R*_s_ = −0.797, *P* < 0.001), as were the Glu/Cr ratio and GluR2 protein level (*R*_s_ = −0.567, *P* = 0.003) (Figures 6(e) and 6(f)). Similarly, the absolute concentration of Glx and the Glx/Cr ratio were negatively correlated with GluR2 expression (*R*_s_ = −0.670, *P* < 0.001; *R*_s_ = −0.476, *P* = 0.016) (Figures 6(g) and 6(h)).

## 4. Discussion

This study investigated the metabolic changes in Glu levels using ^1^H-MRS in vivo and analyzed the role of EAAT2 and GluR2 in regulating the Glu levels during the acute stage of HIBD. Considerable studies have shown that Glu has an important role in maintaining normal brain functions, and Glu concentration in the extracellular fluid must be kept at a low level (<100 *μ*M) to prevent excitotoxicity. Astrocytes play an important role in maintaining Glu homeostasis. Accumulated Glu in the extracellular fluid is mainly taken up by cells via sodium-dependent EAATs. Once inside the astrocytes, Glu is transformed by glutamine synthetase into Gln, which is released from astrocytes. Extracellular Gln is taken up by presynaptic neurons, which complete the Glu-Gln cycle between neurons and astrocytes. HI injury disrupts astrocyte Glu uptake. Thus, HI injury induces extracellular Glu accumulation to high levels, and the resultant excitotoxicity can aggravate brain injury in newborns [21]. Our results are consistent with those of the previous study. The Glu metabolism level sharply increased after HI compared with that in the control group (Figure 5). The degree of injury in newborn piglets became more severe over time after HI insult, indicating that increasing Glu accumulation is closely related to brain damage caused by the resulting excitotoxicity [22]. During the early stage of HI injury, Na^+^/K^+^ pump dysfunction may significantly increase the extracellular K^+^ concentration, promote neuronal depolarization, activate the voltage-dependent calcium channel and massive Ca^2+^ influx, and trigger synaptic terminals to release excessive Glu [15]. During the later stage of HI injury (i.e., 24 h after HI in this study), ATP levels are depleted and reperfusion injury causes cell rupture and/or impaired astrocyte reuptake [23], which again lead to Glu release.

EAAT2 is the primary subtype of EAATs present in the corpus striatum, and EAAT2 in the cell membranes of astrocytes is thought to be responsible for 90% of Glu transport in humans [24], which is crucial for maintaining homeostasis of the Glu-Gln cycle. Therefore, this study focused on changes of EAAT2 after HI. This study demonstrated that Glu levels were significantly increased after HI injury compared with the control, and changes in Glu levels (including the absolute Glu concentration and Glu/Cr ratio) were inversely correlated with changes in EAAT2 expression. This indicates that EAAT2 may have a key role in HI injury by reducing Glu excitotoxicity. EAAT2 expression decreased during early HI injury and subsequently increased, possibly because early HI promoted massive Glu release but inhibited EAAT2 function on the cell membrane and reduced EAAT2 expression. As the HI time increased, EAAT2 expression was elevated, and some Glu underwent oxidative metabolism to provide energy for efficient EAAT2 transport, and Glu depletion reached its peak, suggesting that EAAT2 began to function to prevent massive Glu accumulation. What is more, the protein levels of GFAP increased at this stage; this reactive astrogliosis may be a self-protection mechanism of astrocytes. During a later stage, the expression level of GFAP was still elevated, and this overexpression may be one of the important mechanisms of potential excitotoxicity of neurons. Some studies have also suggested that this overexpression can lead to the formation of glial scars in brain injury areas, which is an important cause of brain nerve regeneration disorders [25]. While the EAAT2 protein expression level declined, the Glu level increased, perhaps because it was difficult to maintain Glu homeostasis due to neuronal necrosis and the functional inhibition of EAAT2 on the astrocyte membrane. Our results confirm that EAAT2 has a critical effect on HIBD. Numerous investigators have tried to reduce HI-induced brain damage by regulating EAAT2 expression. Many current studies that focus on upregulating EAAT2 expression, increasing Glu uptake, reducing Glu excitotoxicity, and relieving nerve injury with resveratrol [26], sulbactam [27], histamine [28], and ceftriaxone [24] confirmed that cerebral ischemic preconditioning could upregulate GLT-1 expression in astrocytes and thus enhance the effects of cerebral ischemic tolerance. At present, some researchers have found that inducing EAAT2 expression in mesenchymal stem cells can significantly reduce glutamate excitotoxicity [29]. Of course, clinical application of these methods requires further validation.

HI-related disruption of the Glu-Gln cycle causes changes in intracellular and extracellular Glu levels. When extracellular Glu reaches a certain level, the activation of related receptors leads to a series of changes, both physiological and pathological. The AMPAR is an important subtype of ionic Glu receptors and is widely expressed in medium spiny neurons of the basal ganglia. AMPAR contains four different subunits, GluR1 to GluR4. GluR2 has been characterized as an important functional moiety of AMPAR. Under normal physiological conditions, GluR2 is highly expressed in AMPAR of most neurons, and it is not permeable to Ca^2+^, which depends on editing of the GluR2 pre-mRNA Q/R (Gln/arginine) site. Changes in GluR2 expression can change Ca^2+^ permeability [9, 12, 13] and thereby play a role in HI-mediated injury. This study evaluated the changes in GluR2 protein levels after HI insult and possible mechanisms mediating HI-induced brain damage. We found that the GluR2 protein levels in the basal ganglia showed a decreasing trend with prolonged HI time. The GluR2 protein levels were negatively correlated with the severity of pathological lesions. We also observed that changes in Glu metabolism levels were negatively correlated with GluR2 protein levels. These results suggest that Glu accumulation after HI leads to the activation of AMPAR and then downregulation of GluR2 expression; what is more, GluR2 expression level further declined as HI injury worsened. Therefore, GluR2 may be involved in the susceptibility of the basal ganglia.

The mechanism obstructing rapid Ca^2+^ influx is markedly weakened after HI-mediated GluR2 decrease, and Ca^2+^ flows into cells via the activated Ca^2+^-permeable AMPAR. This causes intracellular Ca^2+^ overload, which enhances Glu toxicity and causes neuronal death. This mechanism may account for secondary damage to HI. Recent studies showed that GluR2 mRNA expression was downregulated after HI and changes in the functional reactivity of AMPA receptors may mediate Ca^2+^ influx [13]. There was a significant reduction in the GluR2 expression level after reperfusion in the global cerebral ischemia rat model [30], which is consistent with our results. Intracerebral injection of antisense oligonucleotide knocked down GluR2 expression in rats [31], and the death of pyramidal neuronal cells in the hippocampal CA1 region enhanced the pathogenicity of transient ischemic attack.

The mechanisms mediating decreased GluR2 expression after HI remain to be clarified, and several questions remain unanswered. For example, how is GluR2 mRNA transcript editing affected by HI, how do other AMPAR subunits change, and how do the electrophysiological characteristics of AMPAR change? Previous studies suggest that acute downregulation of GluR2 expression can function as a “molecular switch” to form Ca^2+^-permeable AMPAR [9, 13] and strengthen Glu toxicity during nerve injury. Some investigators successfully mitigated or reversed the downregulation of GluR2 expression by treating cells with 3,5,3′-triiodo-L-thyronine (T3) [32], genistein (4′,5,7-trihydroxyisoflavone) [33], or isoflurane [34], thereby protecting neurons from injury induced by Glu excitotoxicity. We used ^1^H-MRS to evaluate the NAA and Cho levels. The results suggest that NAA/Cr ratios gradually declined over time after HI injury and were negatively correlated with the severity of damage to basal ganglia (*R*_s_ = −0.456, *P* = 0.022). Our results were consistent with previous studies [35, 36], which reported that low NAA/Cr indicated poor prognosis after HI. NAA is considered to be a neuronal maker, and NAA levels are closely associated with the number and activity of neurons. The reduction in NAA level after HI is usually irreversible, indicating neuron loss and irreversible brain damage [37]. However, due to the high plasticity of the neonatal brain, differentiation of neuronal stem cells can help to recover damaged neurons in some conditions [38]. Therefore, NAA is not entirely irreversible. Several studies [39, 40] reported that the NAA level was not significantly lower in patients with mild to moderate HIBD but was permanently depleted in those with severe HIBD. Our results were consistent with these studies. During early HI injury, NAA/Cr did not significantly differ from that in the control group. At 72 h after HI injury, the NAA level was significantly lower than that in the control group. This provided further evidence that a permanent depletion of NAA/Cr could be used as an indicator for poor prognosis in HIBD. An interesting thing to note is that our results, different from the previous studies [35, 39, 41], showed that Cho level increased after HI. It may be interpreted as reflecting astrogliosis [42] or deficient development of the neurons [43]. Furthermore, the Cho level correlated positively with the pathological scores in our study (the absolute Cho concentration, *R*_s_ = 0.703, *P* < 0.001; Cho/Cr, *R*_s_ = 0.638, *P* = 0.001). The Cho and Cho/Cr might be used as makers for assessing the degree of brain injury. Future studies with bigger sample sizes need to be conducted to validate this view.

After HI, the immature brain often has a latent period of 8-24 h, which is closely followed by excitotoxicity, inflammation, and oxidative stress response (known as the “deadly triad”) [44, 45]. This can induce secondary energy failure and eventually irreversible neuronal injury. Nerve-protecting strategies must be applied before the occurrence of irreversible injury. The brains of newborn piglets are very similar to those of newborn humans, so newborn piglets were selected as experimental animals in this study. The HI newborn piglet model was used to simulate the pathological process of HIBD in human infants. Histological staining revealed that neuronal injury was not obvious at 6 h after HI, neuronal edema was initially observed at 12 h, and a large number of neurons were edematous at 24 h, but the main pathological change was reversible neuronal necrosis. Neuronal edema became more evident at 72 h, and the brain tissues had irreversible, serious pathological changes (including neuronal apoptosis and necrosis). Our results revealed that there was no irreversible injury and the slight recovery of Glu level within 12 h after HI indicated that this time period before the development of secondary energy failure may be the best time for clinical treatment. In this time window, if drugs or other interventions are used to regulate the expression of EAAT2 and GluR2 proteins to reduce the Glu excitotoxicity, it is expected to open up a new path for pediatricians to carry out timely and effective treatment of HIBD. Notably, the determination of this optimal time window still needs further study.

There are some limitations of note. First, due to the complex of etiology and pathogenesis of HIBD, animal HI models established were different from the clinical cases, which may not accurately display the genesis, development, and pathology of neonatal HIBD. Secondly, a small sample size was used in this study and may suffer from a bias. Further thorough studies in a larger sample are needed to confirm the present results.

## 5. Conclusions

We observed that the Glu levels in the basal ganglia increased after HI and showed a biphasic change. Changes in Glu levels were inversely correlated with changes in EAAT2 and GluR2 expression after HI. The results of this study highlight that ^1^H-MRS can be of use in estimating the activation status of EAAT2 and GluR2 in vivo and provide a reliable imaging evidence for the timely and effective treatment of HIBD. Future studies can focus on reducing excitotoxicity-induced brain damage by regulating the levels of EAAT2 and GluR2 proteins. Because of the particularity of the newborn, more work is needed to be conducted to ensure the safety and effectiveness of clinical medication.

## Figures and Tables

**Table 1:** Pathological scoring of the piglet basal ganglia at different time points after HI treatment.

	Control group (*n* = 5)	HI model group			
		6 h (*n* = 5)	12 h (*n* = 5)	24 h (*n* = 5)	72 h (*n* = 5)
Pathological score	0 (0-0)	1 (1-2)	2 (1.5-2.5)	3 (2.5-3.5)^∗^	4 (3.5-4.5)^∗^

Note: data are displayed as median (25th-75th percentile). ^∗^*P* < 0.05 compared with the control group.

## Data Availability

All data used to support the findings of this study are included within the article.
